# Cyclooxygenase-2 induces neoplastic transformation by inhibiting p53-dependent oncogene-induced senescence

**DOI:** 10.1038/s41598-021-89220-5

**Published:** 2021-05-10

**Authors:** Hyeon Ju Lee, So Ra Kim, Yu-Jin Jung, Jeong A. Han

**Affiliations:** 1 Department of Biochemistry and Molecular Biology, Kangwon National University School of Medicine, Chuncheon, 24341 South Korea; 2 Department of Biological Sciences, Kangwon National University, Chuncheon, South Korea

**Keywords:** Cancer, Oncology

## Abstract

Much in vivo evidence indicates that cyclooxygenase-2 (COX-2) is deeply involved in tumorigenesis. Although it has been proposed that COX-2-derived pro-inflammatory prostanoids mediate the tumorigenic activity of COX-2, the tumorigenic mechanisms of COX-2 are not yet fully understood. Here, we investigated the mechanism by which COX-2 causes transformation from normal cells to malignant cells by using normal murine or human cells. We found that COX-2 inhibits the pro-senescent function of p53 under oncogenic RAS activation, by which it prevents oncogene-induced senescence (OIS) and induces neoplastic transformation. We also found that COX-2 physically interacts with p53 in the nucleus under oncogenic RAS activation, and that this COX-2-p53 interaction rather than the catalytic activity is involved in the COX-2-mediated inhibition of the pro-senescent function of p53 and OIS, and induction of neoplastic transformation. These findings strongly suggest that the oncogenic property of COX-2 is closely related to its ability to inactivate p53 under strong mitogenic signals, and that aberrant activation of the COX-2/a mitogenic oncogene combination can be a potent driving force for tumorigenesis. This study might contribute to our understanding of the molecular basis for the tumorigenic activity of COX-2 and the development of novel anti-tumor drugs targeting COX-2-p53 interactions.

## Introduction

Malignant transformation is the process by which normal cells acquire the traits of cancer cells. Sustained mitogenic signaling is the fundamental trait of cancer cells, which is acquired by oncogenic activation of mitogenic proto-oncogenes^1,2^. In normal cells, however, strong mitogenic signals elicited by activated oncogenes do not lead to malignant transformation but often lead to permanent cell cycle arrest called oncogene-induced senescence (OIS). Many proto-oncogenes including *RAS, RAF, MOS, ERK, AKT, CDC6, CYCLIN E* or *E2F1* have been shown to induce OIS when overexpressed or expressed as oncogenic forms^3–6^. In cultured cells and model animals, it is well known that overcoming OIS is a prerequisite for malignant transformation^7–9^. In humans, senescent cells are more predominantly observed in precancerous lesions than in normal and cancer tissues^10^. Therefore, OIS is thought to be a crucial barrier against malignant transformation in vitro as well as in vivo^6–10^.

OIS is known as a cellular response to DNA damage, where the p53/p21^CIP1/WAF1^ pathway plays a central role. The sustained and strong mitogenic signals by activated oncogenes cause replication stress in DNA, which in turn leads to DNA double-strand breaks (DSBs). This triggers the DNA damage response (DDR) that involves the sequential activation of ATM, Chk2 and p53^10–13^. Activated p53, a transcription factor, moves from the cytosol to the nucleus, where it promotes the transcription of a pro-senescent target gene *p21*^*CIP1/WAF1*^. p21^CIP1/WAF1^, a crucial mediator of the pro-senescent function of p53, is a classic cyclin-dependent kinase (Cdk) inhibitor, whose senescence mechanism is currently unclear^13,14^.

p53, the most frequently inactivated tumor suppressor in human cancers, strongly prevents malignant transformation in vitro and in vivo. p53 inactivation is sufficient to overcome OIS and induce malignant transformation in normal rodent cells and in mice under strong mitogenic signals^3,7–9^. In normal human cells, p53 inactivation does or does not prevent OIS in the cellular context^3,11,12,15^, and additional factors such as activation of telomerase, inactivation of the p16/pRb pathway and perturbation of PP2A are required to induce malignant transformation^7,8^.

Prostaglandin-endoperoxide synthase (PTGS), also called as cyclooxygenase (COX), is an enzyme that catalyzes the conversion from arachidonic acid to prostaglandin H_2_ (PGH_2_). PGH_2_ is then converted to various prostanoids such as PGE_2_, PGI_2_, PGF_2α_, PGD_2_ and thromboxane A_2_ (TXA_2_), which are important mediators of inflammation^16^. Of the two isozymes of COX, COX-1 is known as a constitutive form, whereas COX-2 is an inducible form whose expression is induced by various stimuli^16^. Most non-steroidal anti-inflammatory drugs (NSAIDs) target the COX catalytic activity. Non-selective COX inhibitors including aspirin, ibuprofen, piroxicam and sulindac inhibit both COX-1 and COX-2 catalytic activity, whereas selective COX-2 inhibitors including celecoxib, nimesulide and NS-398 inhibit the COX-2 catalytic activity preferentially^17^.

Many in vivo data indicate that COX-2 is deeply involved in the entire stages of tumorigenesis—initiation, promotion and progression^18,19^. For example, COX-2 is overexpressed in precancerous lesions as well as in cancer tissues compared to normal tissues^20,21^. In addition, COX-2 overexpression caused dysplasia and hyperplasia in the mammary glands, urinary bladder and pancreas of the transgenic mice, which further developed into metastatic malignant tumors^22–24^. Moreover, *COX-2* knockouts prevented the formation of benign or malignant tumors in the skin of ultraviolet B (UVB)-irradiated mice and intestines of *APC*^*Δ*716^ mice, a model of familiar adenomatous polyposis (FAP)^25–27^. Furthermore, NSAIDs prevented the formation of aberrant crypt foci (ACF), benign tumors, and malignant tumors in the colons of azoxymethane (AOM)-injected rats^28–31^. Of note, NSAIDs more effectively prevented the benign and malignant tumor formation when administered in the initiation phase than in the promotion/progression phase in AOM-injected rats^28,30^. NSAIDs have also been shown to prevent the formation of benign and malignant tumors, or metastasis in various human organs^32^.

It has been proposed that COX-2-derived pro-inflammatory prostanoids, especially PGE_2_, mediate the tumorigenic activity of COX-2^19,33,34^. However, the molecular mechanisms by which COX-2 promotes tumorigenesis, especially in the early stages of tumorigenesis, are largely unknown. In fact, cellular studies have shown unexpected results. Overexpression of COX-2 induced cell cycle arrest, not malignant transformation, in not only normal cells such as bovine endothelial cells and rat mesangial cells, but also in immortalized cell lines including ECV304, HEK293, COS-7 and NIH3T3, in a catalytic activity-independent manner^35,36^. Also, COX-2 overexpression failed to induce foci formation on the soft agar in MCF-10F and SV-HUC immortalized cells^37,38^. In addition, embryonic fibroblasts from *COX-2*(+/+) and *COX-2*(−/−) mice were readily transformed with equal efficiency by *EJ-ras/SV40*, a known inducer of malignant transformation^39^.

Here we show a novel mechanism by which COX-2 contributes to tumorigenesis. Specifically, we found that COX-2 prevents OIS under strong mitogenic signals, by which it induces neoplastic transformation in normal cells. We also found that the COX-2-mediated prevention of OIS and induction of neoplastic transformation is attributable to the COX-2-mediated inhibition of the pro-senescent function of p53 by protein–protein interactions.

## Materials and methods

### Cell culture

Mouse embryonic fibroblasts (MEFs) were isolated from pregnant ICR mice^40^. Human diploid fibroblasts (HDFs) from foreskin were obtained from Pf. S. C. Park (Chonnam National University)^41^. Cells were maintained in Dulbecco’s Modified Eagle’s Medium (DMEM) containing 10% fetal bovine serum (#F60001, Median Life Science, USA), penicillin (100 units/ml) and streptomycin (100 units/ml) in a 5% CO_2_ incubator.

### Plasmids and retroviruses

cDNAs of human COX-2, the S516Q mutant of COX-2, (myc)_6_-tagged COX-2 deletion mutants, p53, H-RAS^V12^ and SV40 LT were subcloned into the pMSCVpuro vector (Clontech). Cloned plasmids were transfected into H29D packaging cells using polyethylenimine^42^. Conditioned media were collected from 2 to 4 days after the transfection and centrifuged. Cells were added with the supernatant containing retroviruses in the presence of 2 μg/ml polybren and incubated for 24 h for each kind of retroviruses. After maintenance in the normal media for 2 days, cells were selected in the presence of 1 μg/ml puromycin for 2 days. After stabilization in the normal media for 2 days, cells were seeded for various assays.

### SA-β-gal staining

Staining for the senescence-associated β-galactosidase (SA-β-gal) activity was carried out at pH 6.0 as described. Briefly, cells were fixed with 3% formaldehyde for 5 min, and stained at 37 °C in a solution containing 40 mM citric acid/20 mM sodium phosphate, pH 6.0, 150 mM NaCl, 2 mM MgCl_2_, 5 mM potassium ferricyanide, 5 mM potassium ferrocyanide and 1 mg/ml X-gal^41^. After 24 h, 100–200 cells per sample were examined and the percentage of stained cells was calculated.

### PGE_2_ assay

The amount of PGE_2_ secreted into the culture medium was determined by using an enzyme-linked immunosorbent assay kit according to the manufacturer’s instruction (#K018-H1, Arbor Assays, USA).

### Western blot analysis

Cells were lysed in a lysis buffer (50 mM Tris–HCl, pH 7.4, 150 mM NaCl, 1% NP-40, 0.25% sodium deoxycholate and 0.1% SDS) with a protease inhibitor cocktail (#P2714, Sigma-Aldrich). Proteins were resolved on SDS–polyacrylamide gels and transferred to PVDF membranes, which were then probed with specific antibodies. The protein-antibody complexes were detected by enhanced chemiluminescence (#LF-QC0103, AbFrontier, South Korea). Specific antibodies for COX-2 (1:500; #sc-1745), p21 (1:500; #sc-397), p16 (1:50; #sc-28260) and β-actin (1:20,000; #sc-81178) were purchased from Santa Cruz Biotechnology. Antibodies for p53 (1:500; #2524) and Ras (1:1000; #OP40) were purchased from Cell Signaling Biotechnology and Calbiochem, respectively. Blots were cut prior to hybridization with the specific antibodies and therefore the whole length blots were not provided.

### Immunoprecipitation

Cells were lysed in a lysis buffer (50 mM Tris–HCl, pH 7.4, 150 mM NaCl, 1% NP-40, 0.25% sodium deoxycholate, 0.1% SDS, 50 mM NaF and 1 mM Na_3_VO_4_) with a protease inhibitor cocktail. Cell lysates were mixed with 5 ul of an anti-COX-2 polyclonal antibody (#sc-1745, Santa Cruz Biotechnology) or an anti-p53 monoclonal antibody (#2524, Cell Signaling Biotechnology) at 4 °C overnight. Then, it was mixed with 20 ul of Protein A/G PLUS-agarose beads (#sc-2003, Santa Cruz Biotechnology) for 2 h at 4 °C. The beads were collected, and the bead-bound proteins were subjected to western blot analysis using the anti-p53 monoclonal antibody (1:500) and the anti-COX-2 polyclonal antibody (1:500), respectively.

### Confocal microscopy

Cells were seeded on 18 mm coverslips and cultured overnight. Cells were then fixed with 3.7% formamide and permeabilized with 0.2% Triton X-100. After blocking with 2% bovine serum albumin, cells were incubated with both anti-COX-2 polyclonal antibody (1:100; #sc-1745, Santa Cruz Biotechnology) and anti-p53 monoclonal antibody (1:100; #2524, Cell Signaling Biotechnology, 1:100) at 4 °C overnight. Cells were then incubated with both TRITC-conjugated anti-goat IgG (1:200; #A-21432, Thermo Fisher Scientific) and FITC-conjugated anti-mouse IgG (1:200; #F6257, Sigma-Aldrich) for 1 h at room temperature. Cell images were taken with a confocal microscope (Carl Zeiss).

### Soft agar assay

The 2.5 × 10^4^ cells were resuspended in the culture medium containing 0.35% low-melting agarose (#A4018, Sigma-Aldrich). Then, the cells were overlaid on top of 0.5% solidified agarose in 6-well plates. After about 4 weeks, the total number of foci per well was counted under a light microscope.

### In vivo tumor formation assay

All procedures were approved by the Institutional Animal Care and Use Committee of the Kangwon National University (#KIACUC-11-0006) and carried out in compliance with the ARRIVE guidelines (https://arriveguidelines.org/). Female BALB/c nude mice at 6 weeks of age were obtained from Nara Biotech (South Korea). Five to seven mice were allocated to each group and then injected subcutaneously in each flank with 3 × 10^6^ MEFs in 100 μl of PBS. Tumor volumes were measured with an electronic caliper every 2–3 days and calculated with the formula x^2^ x y/2, where x is the width and y is the length^43^. All experiments were performed in accordance with relevant guidelines and regulations.

### Statistical analysis

Unless otherwise noted, we performed a nonparametric Kruskal–Wallis test on the entire data set and then performed a Wilcoxon rank sum test on the specific two groups of interest by using the RStudio program (version 1.2.1355 downloaded from https://www.rstudio.com). Differences between groups were considered statistically significant when the *P* values were < 0.05.

## Results

### COX-2 overexpression prevents OIS under oncogenic RAS activation

**Figure 1:**
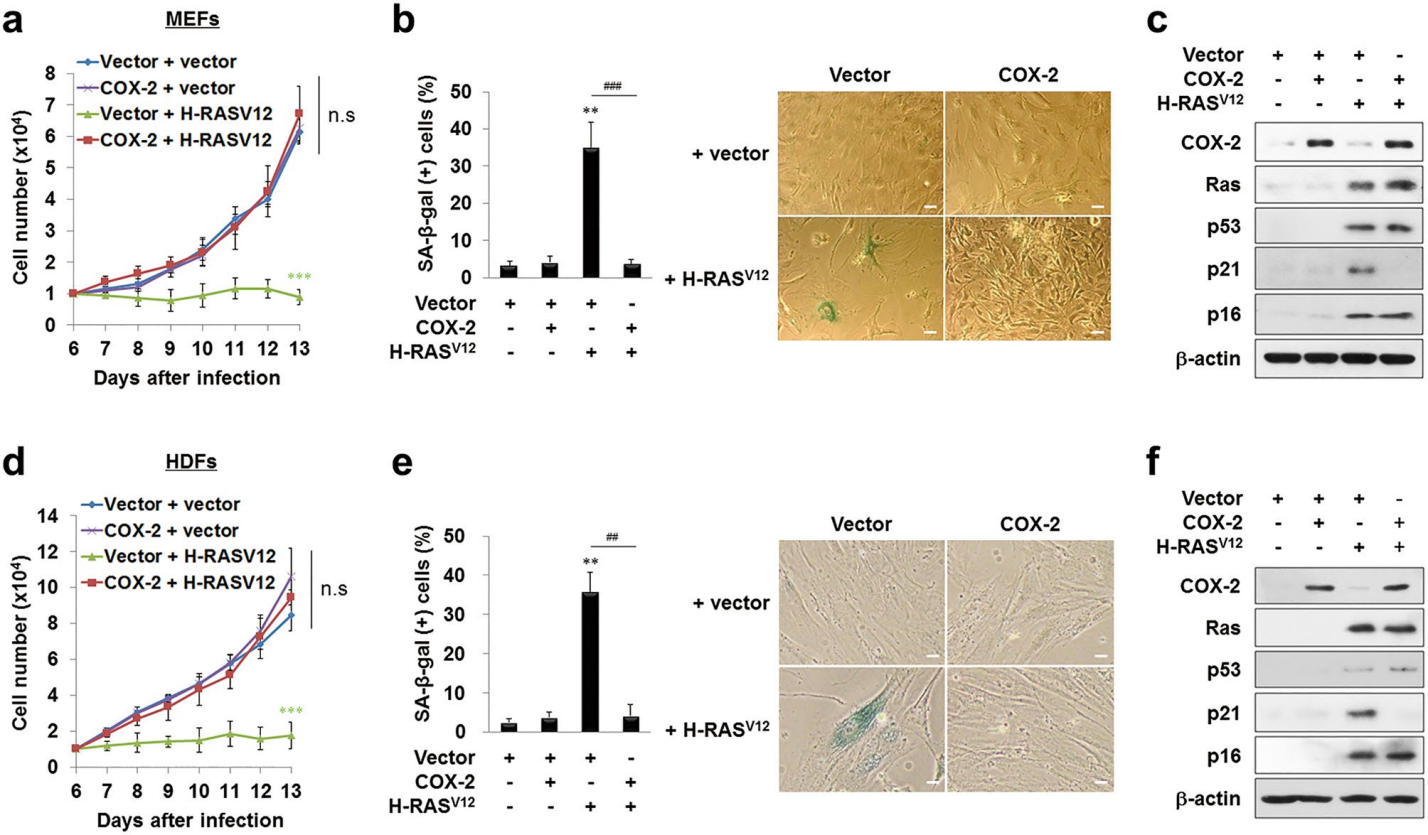
COX-2 overexpression prevents OIS under oncogenic RAS activation in MEFs (**a**–**c**) and HDFs (**d**–**f**). (**a**,**d**) Cells were infected with retroviruses as indicated. 1 × 10^4^ cells were seeded and the number of cells was counted. Data are means ± SD (*n* = 11) in (**a**) and SE (*n* = 6) in (**d**) (***P < 0.001 vs. Vector + vector; n.s, not significant at the final day). (**b,e**) The ratios of SA-β-gal (+) cells were calculated at 14 days after the infection (left). Representative staining data were shown (right). Data are means ± SD (*n* = 9 in (**b**) and *n* = 6 in (**e**)) (**P < 0.01 vs. Vector; ^##^*P* < 0.01, ^###^*P* < 0.001). Scale bars = 13.6 μm. (**c**,**f**) Western blot analysis was performed using cell lysates. β-actin was used as a loading control.

To elucidate the mechanism by which COX-2 causes transformation from normal cells to malignant cells, we first investigated the cellular function of COX-2 under strong mitogenic signals in normal cells. For this purpose, we infected early-passage mouse embryonic fibroblasts (MEFs) with retroviruses expressing H-RAS^V12^, an oncogenic form of RAS, with or without COX-2. MEFs transfected with H-RAS^V12^ alone showed growth arrest and increased the ratios of senescence-associated β-galactosidase (SA-β-gal) (+) cells, indicating that H-RAS^V12^ expression induced senescence as described^3^. In contrast, MEFs transfected with both COX-2 and H-RAS^V12^ proliferated well and showed a basal level of SA-β-gal (+) cell ratios (Fig. 1a,b). These results suggest that COX-2 overexpression prevents OIS under strong mitogenic signals in normal murine cells.

To examine the role of COX-2 in normal human cells, we transfected human diploid fibroblasts (HDFs) with H-RAS^V12^ with or without COX-2. HDFs transfected with H-RAS^V12^ showed premature growth arrest and increased SA-β-gal (+) cell ratios. In contrast, HDFs transfected with both COX-2 and H-RAS^V12^ proliferated well and showed a basal level of SA-β-gal (+) cell ratios (Fig. 1d,e). These results suggest that COX-2 overexpression prevents OIS under strong mitogenic signals in normal human cells, too.

### COX-2 overexpression prevents OIS by inhibition of the pro-senescent function of p53

The H-RAS^V12^ transfection in MEFs and HDFs was observed to increase the expression of senescence-associated genes, *p53*, *p21*^*CIP1/WAF1*^ and *p16*, compared to the Vector transfection as described^3^. Among these genes, the expression levels of p53 and p16 were not altered by COX-2 co-transfection in both cells. Intriguingly, however, the expression levels of *p21*^*CIP1/WAF1*^—the crucial pro-senescent target gene of p53—were decreased by COX-2 co-transfection in both cells (Fig. 1c,f). These results suggest that COX-2 overexpression prevents OIS by inhibiting the pro-senescent function of p53 under strong mitogenic signals.

**Figure 2:**
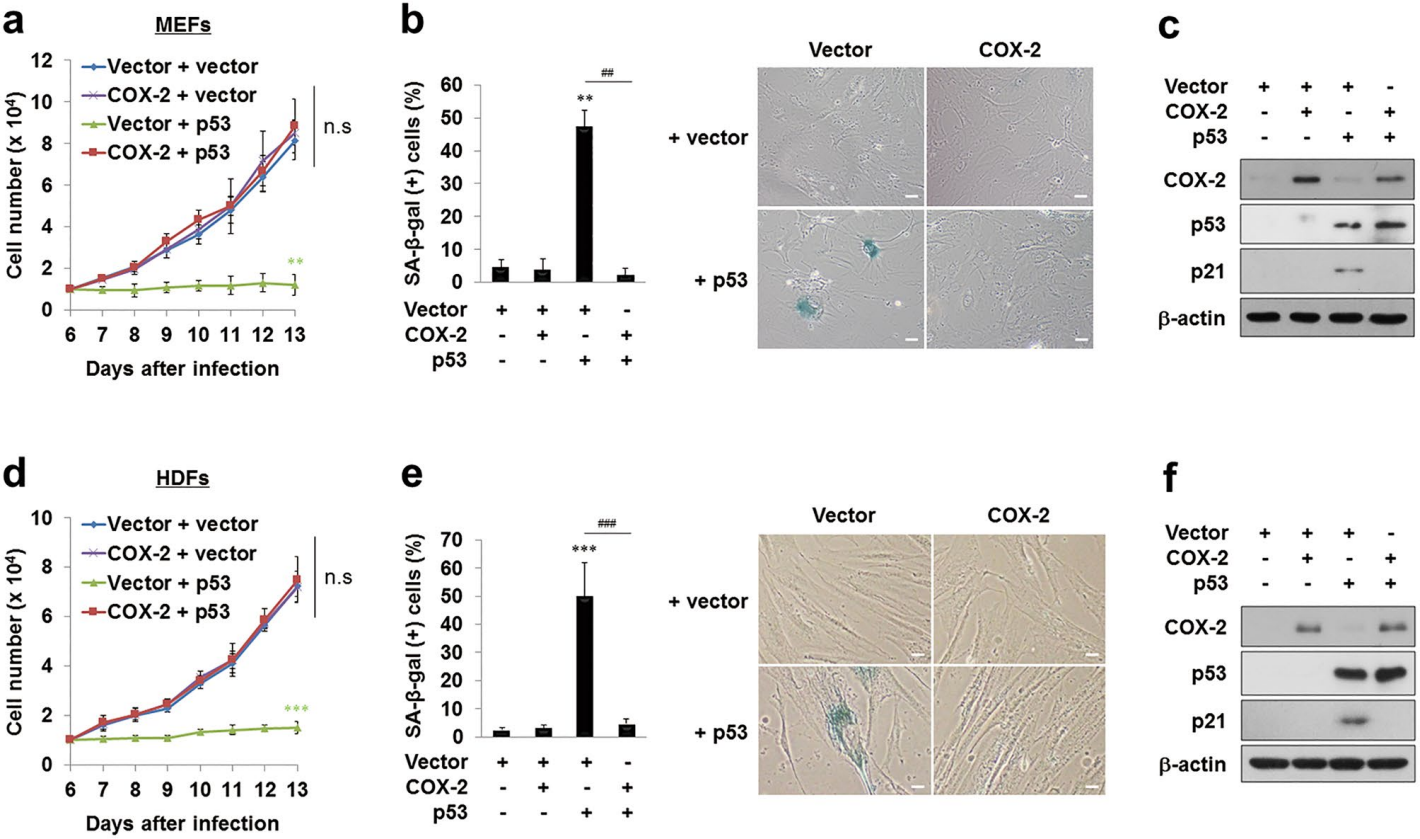
COX-2 overexpression inhibits the pro-senescent function of p53 in MEFs (**a**–**c**) and HDFs (**d**–**f**). (**a**,**d**) Cells were infected with retroviruses as indicated. 1 × 10^4^ cells were seeded and the number of cells was counted. Data are means ± SD (*n* = 6) in (**a**) and SE (*n* = 9) in (**d**) (**P < 0.01, ***P < 0.001 vs. Vector + vector; n.s, not significant at the final day). (**b,e**) The ratios of SA-β-gal (+) cells were calculated at 14 days after the infection (left). Representative data were shown (right). Data are means ± SE (*n* = 6) in (**b**) and SD (*n* = 13) in (**e**) (**P < 0.01, ***P < 0.001 vs. Vector; ^##^*P* < 0.01, ^###^*P* < 0.001). Scale bars = 13.6 μm. (**c,f**) Western blot analysis was performed using cell lysates. β-actin was used as a loading control.

To verify the inhibitory effect of COX-2 on the pro-senescent function of p53, we examined the role of COX-2 in p53-induced senescence. Early-passage MEFs and HDFs infected with retroviruses expressing p53 showed growth arrest and increased SA-β-gal (+) cell ratios as expected. In contrast, MEFs and HDFs transfected with both COX-2 and p53 proliferated well and showed a basal level of SA-β-gal (+) cell ratios (Fig. 2a,b,d,e). In addition, the p53 transfection induced p21^CIP1/WAF1^ expression in MEFs and HDFs, which was prevented by COX-2 co-transfection in both cells (Fig. 2c,f). These results confirm the inhibitory effect of COX-2 on the pro-senescent function of p53, supporting our conclusion that COX-2 overexpression prevents OIS by inhibiting the pro-senescent function of p53.

### COX-2 overexpression induces neoplastic transformation under oncogenic RAS activation

**Figure 3:**
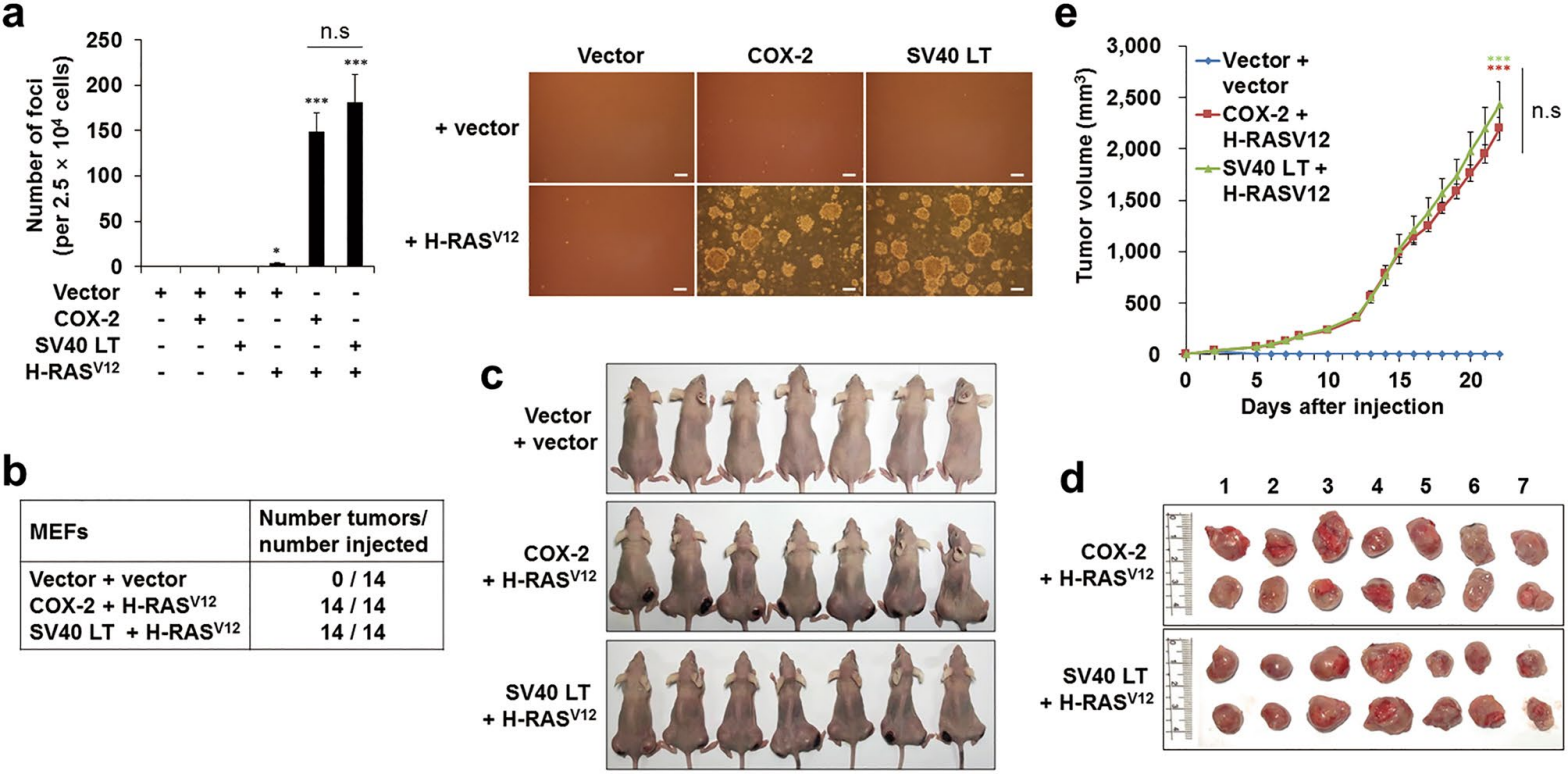
COX-2 overexpression induces neoplastic transformation under oncogenic RAS activation in MEFs. (**a**) Soft agar assay. MEFs were infected with retroviruses as indicated. 2.5  ×  10^4^ cells were seeded on the soft agar and the number of foci was counted (left). Representative data were shown (right). Data are means ± SE (*n* = 8; *P < 0.05, ***P < 0.001 vs. Vector; n.s, not significant). Scale bars = 100 μm. (**b**–**e**) In vivo tumor formation assay. MEFs were infected with retroviruses and injected into nude mice. The total number of tumors per group (**b**), mice (**c**), resected tumors (**d**) and tumor volumes (**e**) were shown. Data are means ± SE (*n* = 14; ***P < 0.001 vs. Vector + vector; n.s, not significant at the final day).

The escape from OIS is known to be sufficient to induce malignant transformation in normal rodent cells^6–8^. Therefore, we next investigated whether COX-2 overexpression could induce transformation in MEFs. Like MEFs transfected with both simian virus 40 large T (SV40 LT) and H-RAS^V12^, a known genetic combination for malignant transformation^7,8,39^, MEFs transfected with both COX-2 and H-RAS^V12^ showed faster proliferation rates and altered morphology compared to MEFs transfected with the Vector (Supplementary Fig. S1). In addition, MEFs transfected with COX-2 or H-RAS^V12^ rarely formed foci on the soft agar, whereas MEFs transfected with both COX-2 and H-RAS^V12^ formed foci easily on the soft agar like the SV40 LT/H-RAS^V12^-transfected MEFs (Fig. 3a). Moreover, the COX-2/H-RAS^V12^-transfected MEFs formed tumors easily in all injected sites of nude mice like the SV40 LT/H-RAS^V12^-transfected MEFs (Fig. 3b–e). These results suggest that COX-2 overexpression can induce neoplastic transformation in normal cells under strong mitogenic signals.

### COX-2 inhibits the pro-senescent function of p53 and OIS by catalytic activity-independent mechanisms

**Figure 4:**
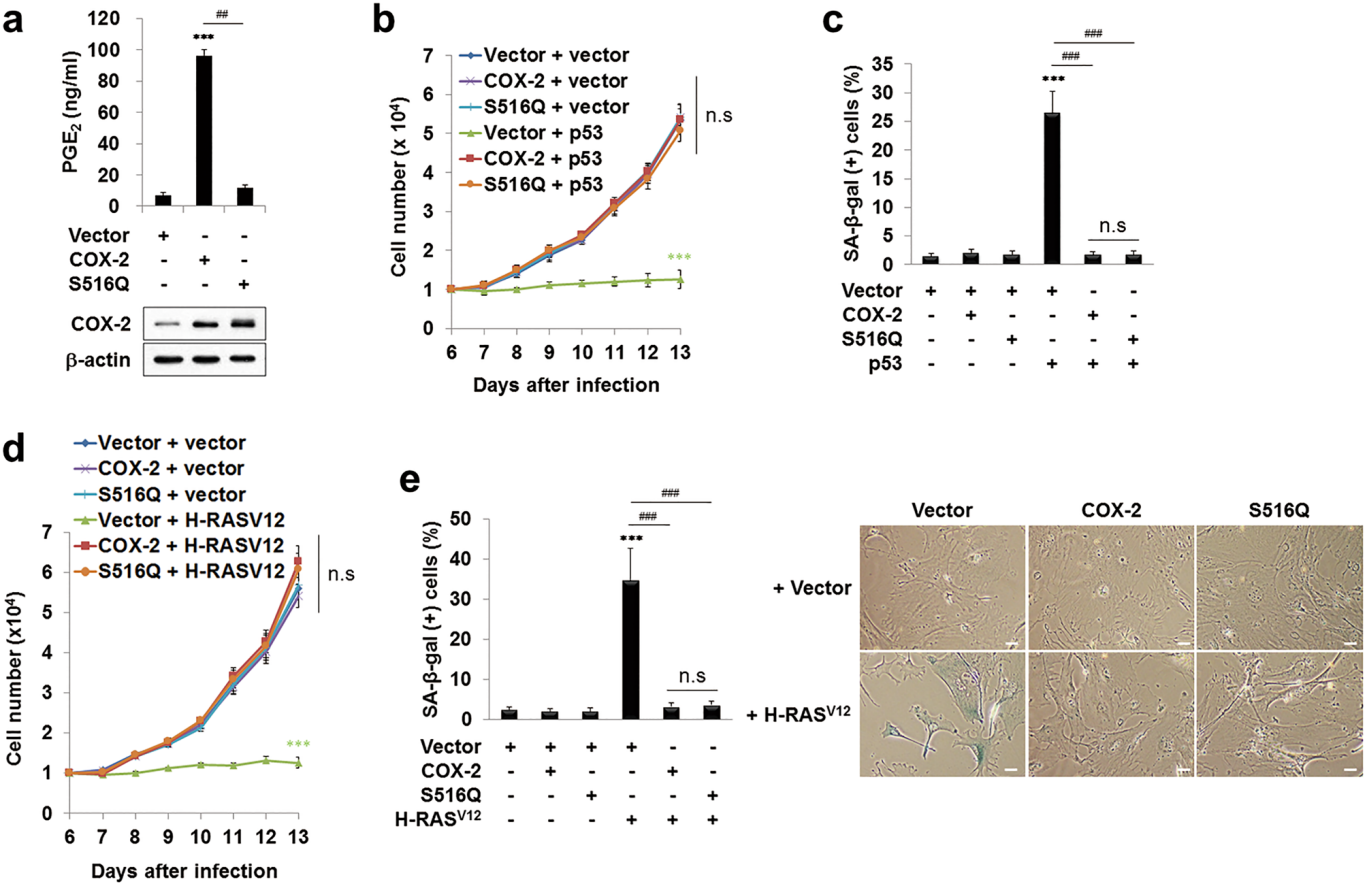
COX-2 inhibits the pro-senescent function of p53 and OIS by catalytic activity-independent mechanisms. (**a**) PGE_2_ assay. MEFs were infected with retroviruses expressing COX-2, S516Q, or an empty vector as a control. PGE_2_ concentration was measured in the conditioned medium (upper). Cell lysates were subjected to western blot analysis. β-actin was used as a loading control (lower). Data are means ± SD (*n* = 6; ***P < 0.001 vs. Vector; ^##^*P* < 0.01). (**b**,**d)** MEFs were infected with retroviruses as indicated. 1  ×  10^4^ cells were seeded and the number of cells was counted. Data are means ± SD (*n* = 6) in (**b**) and SE (*n* = 11) in (**d**) (***P < 0.001 vs. Vector + vector; n.s, not significant at the final day). (**c**,**e**) SA-β-gal (+) cell ratios were calculated and the representative data were shown. Data are means ± SD (*n* = 12 in (**c**) and *n* = 9 in (**e**)) (***P < 0.001 vs. Vector; ^###^*P* < 0.001; n.s, not significant). Scale bars = 13.6 μm.

We next investigated the mechanism by which COX-2 inhibits the pro-senescent function of p53. As COX-2 is an enzyme, we first tested the possibility that the catalytic activity is involved. For this purpose, we generated retroviruses expressing an active site mutant of COX-2, S516Q, and compared the effect of wild-type COX-2 and the S516Q mutant on the p53-induced senescence. The wild-type COX-2 increased PGE_2_ production in MEFs, whereas the S516Q mutant did not increase the PGE_2_ production compared to the Vector control (Fig. 4a), confirming that the S516Q mutant is catalytically inactive as reported^35,44^. The p53 transfection in MEFs induced growth arrest and SA-β-gal activities, which was prevented by both wild-type COX-2 and the S516Q mutant with equal efficiency (Fig. 4b,c). These results suggest that the COX-2 catalytic activity is not involved in the COX-2-mediated inhibition of pro-senescent function of p53, indicating that COX-2 inhibits the pro-senescent function of p53 by catalytic activity-independent mechanisms.

To confirm the efficacy of wild-type COX-2 and the S516Q mutant on the pro-senescent function of p53, we examined their effect on H-RAS^V12^-induced senescence. We observed that both wild-type COX-2 and the S516Q mutant prevented the H-RAS^V12^-induced growth arrest and SA-β-gal activities in MEFs. Again, there was no significant difference between the wild-type COX-2 and the S516Q mutant in the inhibitory effect on H-RAS^V12^-induced growth arrest and SA-β-gal activities (Fig. 4d,e). These results suggest that the COX-2 catalytic activity is not involved in the COX-2-mediated prevention of OIS, supporting our conclusion that COX-2 inhibits the pro-senescent function of p53 by catalytic activity-independent mechanisms.

### COX-2 inhibits the pro-senescent function of p53 and OIS by physical interactions with p53

**Figure 5:**
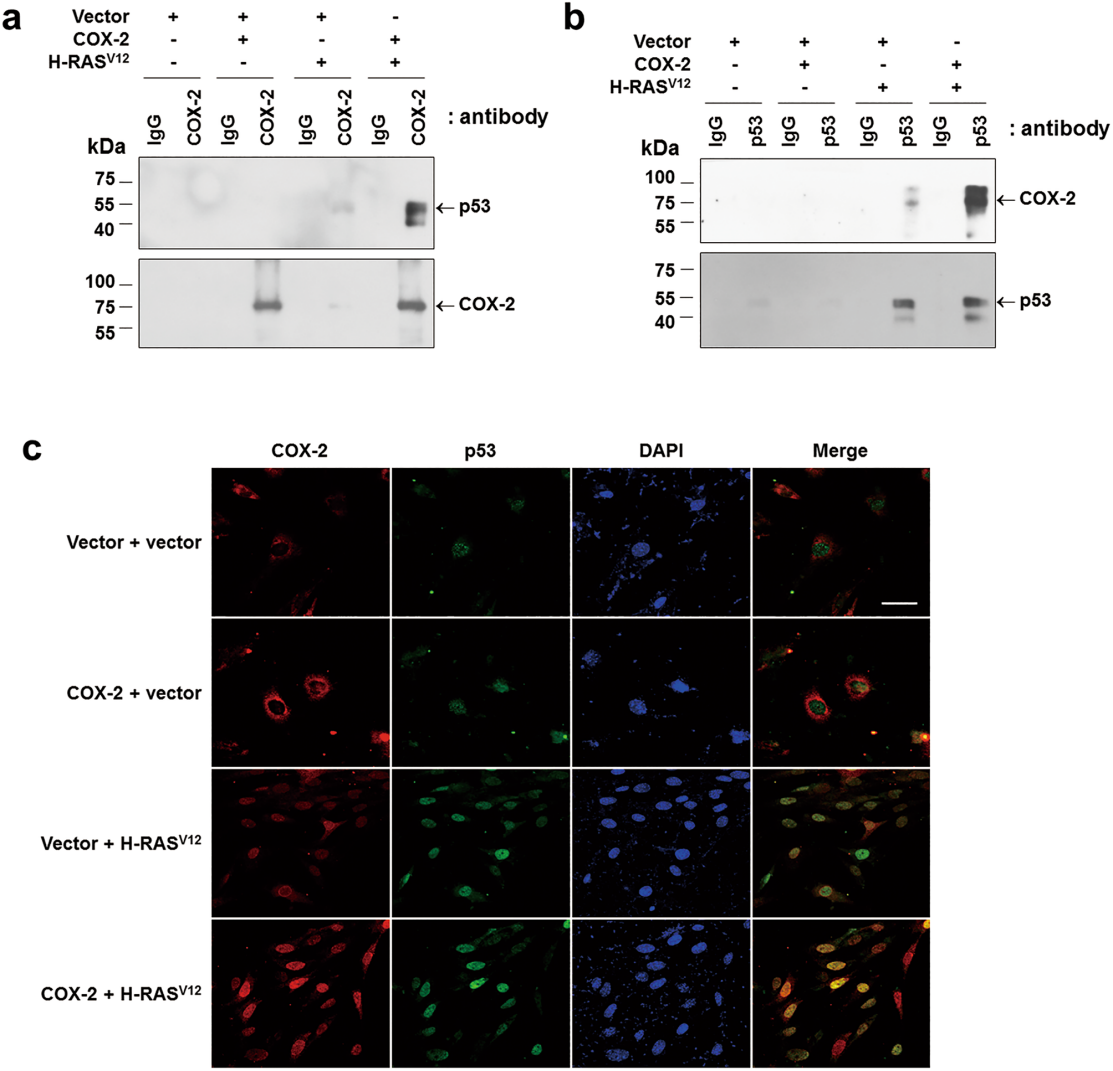
COX-2 interacts with p53 in the nucleus under oncogenic RAS activation. (**a**, **b**) MEFs were infected with retroviruses as indicated. Cell lysates were subjected to immunoprecipitation assays using an anti-COX-2 antibody (**a**) and an anti-p53 antibody (**b**). (**c**) Confocal microscopy was performed using an anti-COX-2 antibody (red), an anti-p53 antibody (green), and DAPI (blue). Merged images of red and green channels were also shown. Scale bar = 50 μm.

In regard to the catalytic activity-independent mechanisms, it has been reported that COX-2 physically interacts with p53 through its amino-terminal region (amino acids 1–126)^44,45^. This led us to hypothesize that COX-2 might inhibit the pro-senescent function of p53 through physical interactions with p53. To test this hypothesis, we first examined whether COX-2 interacts with p53 under strong mitogenic signals by using immunoprecipitation assays. The data showed that, under H-RAS^V12^ transfection, p53 and COX-2 were co-precipitated by an anti-COX-2 antibody and an anti-p53 antibody, respectively, and that the amount of precipitated p53 and COX-2 was dramatically increased by COX-2 co-transfection in MEFs (Fig. 5a, b). These results suggest that COX-2 physically interacts with p53 under strong mitogenic signals, and that overexpression of COX-2 inhibits the pro-senescent function of p53 and OIS by physical interactions with p53.

To determine the location of the COX-2-p53 interaction, we performed a confocal microscopy in MEFs. In resting states, COX-2 was observed in the perinuclear region, while p53 was observed in the nucleus. Upon H-RAS^V12^ transfection, however, COX-2 was observed to translocate to the nucleus, showing that COX-2 co-localized with p53 in the nucleus (Fig. 5c). These results suggest that COX-2 interacts with p53 in the nucleus under strong mitogenic signals, and that COX-2 overexpression inhibits the pro-senescent function of p53 and OIS by physical interactions with p53 in the nucleus.

**Figure 6:**
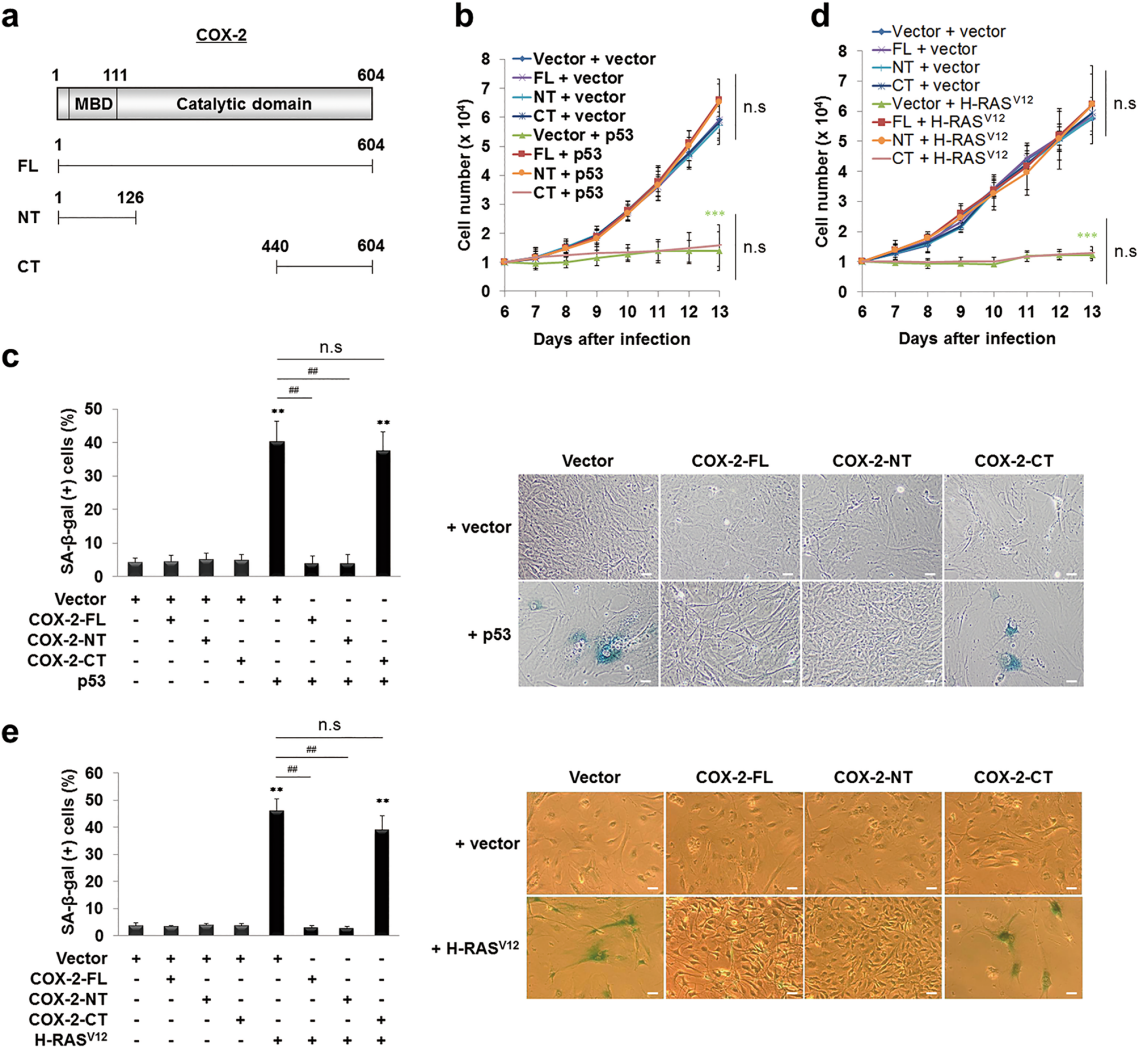
COX-2 inhibits the pro-senescent function of p53 and OIS by physical interactions with p53. (**a**) Schematic representation of the structure of human COX-2 and its deletion mutants (MBD, the membrane-binding domain). (**b**,**d**) MEFs were infected with retroviruses as indicated. 1  ×  10^4^ cells were seeded and the number of cells was counted. Data are means ± SD (*n* = 10 in (**b**) and *n* = 8 in (**d**)) (***P < 0.001 vs. Vector + vector; n.s, not significant at the final day). (**c**,**e**) SA-β-gal (+) cell ratios were calculated (left) and the representative data were shown (right). Data are means ± SE (*n* = 12; **P < 0.01 vs. Vector; ^##^*P* < 0.01; n.s, not significant). Scale bars = 13.6 μm.

To clarify whether the COX-2-p53 interaction is involved in the COX-2-mediated inhibition of pro-senescent function of p53, we generated retroviruses expressing (myc)_6_-tagged COX-2 deletion mutants containing the full-length (FL, amino acids 1–604), the p53-interacting region (NT, amino acids 1–126), and a carboxy-terminal region (CT, amino acids 440–604) as a negative control (Fig. 6a). Then, we assessed the effect of myc-COX-2 constructs on the p53-induced senescence. The data showed that p53 transfection induced growth arrest and SA-β-gal activities in MEFs, which was completely inhibited by COX-2-FL and -NT, but not by COX-2-CT (Fig. 6b,c). These results demonstrate that the p53-interacting region of COX-2 is critical to inhibit the pro-senescent function of p53, strongly suggesting that COX-2 inhibits the pro-senescent function of p53 by physical interactions with p53.

To confirm the effect of myc-COX-2 constructs on the pro-senescent function of p53, we monitored the effect of myc-COX-2 constructs on H-RAS^V12^-induced senescence. The data showed that H-RAS^V12^ transfection induced growth arrest and SA-β-gal activities in MEFs, which was completely prevented by COX-2-FL and -NT, but not by COX-2-CT (Fig. 6d,e). In addition, the H-RAS^V12^ transfection induced p21^CIP1/WAF1^ expression, which was prevented by COX-2-FL and -NT, but not by COX-2-CT (Supplementary Fig. S2). These results suggest that the COX-2-p53 interaction is involved in the COX-2-mediated prevention of OIS, supporting our conclusion that COX-2 inhibits the pro-senescent function of p53 by physical interactions with p53.

### The COX-2-p53 interaction, not the catalytic activity, is involved in the COX-2-mediated neoplastic transformation

**Figure 7:**
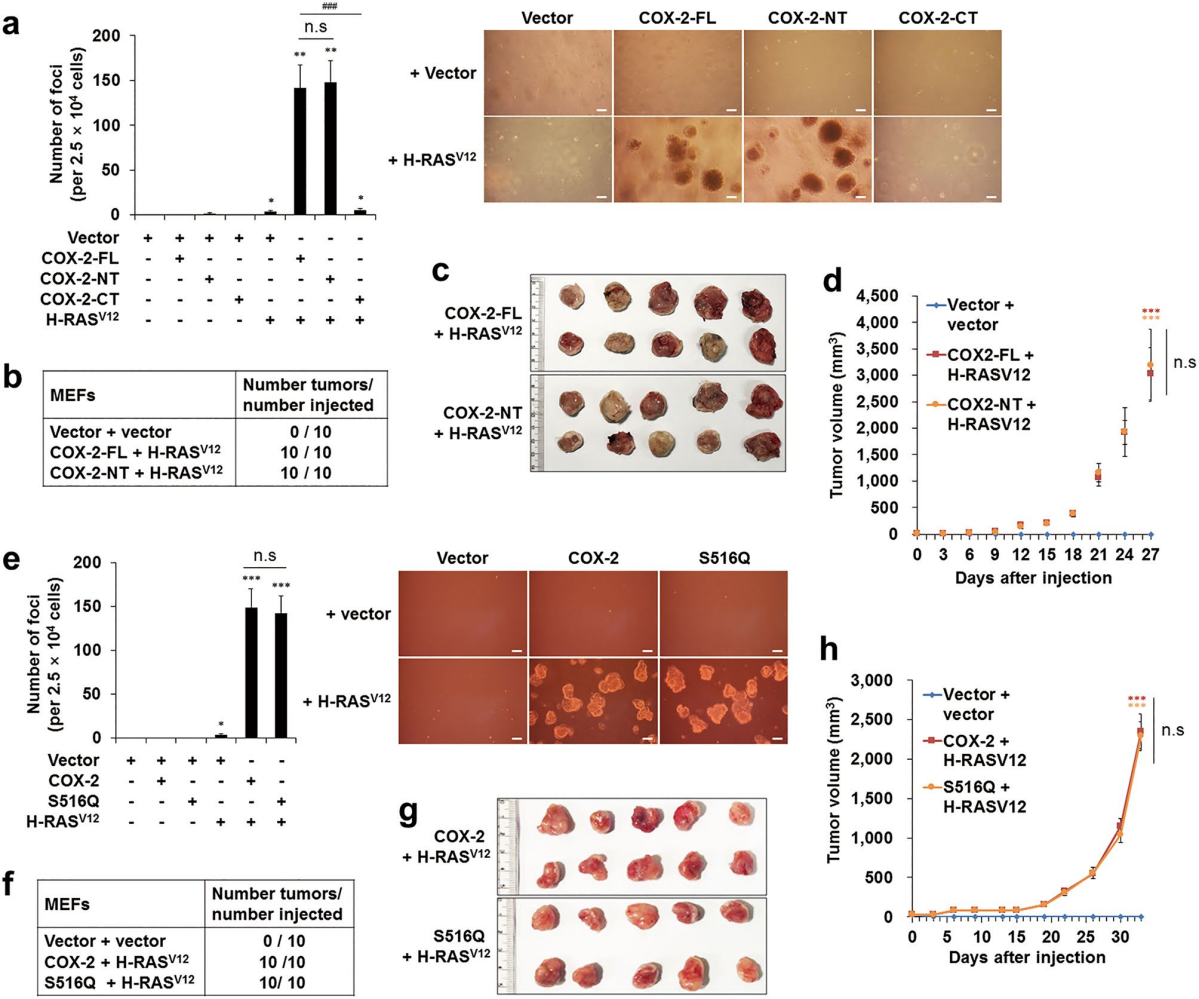
The COX-2-p53 interaction, not the catalytic activity, is involved in the COX-2-mediated neoplastic transformation. (**a**,**e**) Soft agar assay. MEFs were infected with retroviruses as indicated. Cells were seeded on the soft agar and the number of foci was counted (left). Representative data were shown (right). Data represent means ± SE (*n* = 8; *P < 0.05, **P < 0.01, ***P < 0.001 vs. Vector; ^###^*P* < 0.001; n.s, not significant). Scale bars = 100 μm. (**b**–**d**,**f**–**h)** In vivo tumor formation assay. MEFs were infected with retroviruses as indicated and injected into nude mice. Total number of tumors per group (**b**,**f**), resected tumors (**c**,**g**) and tumor volumes (**d**,**h**) were shown. Data represent means ± SE in (**d**) and SD in (**h**) (*n* = 10; ***P < 0.001 versus Vector + vector; n.s, not significant at the final day).

Since our data indicate that the COX-2-p53 interaction is responsible for the COX-2-mediated inhibition of pro-senescent function of p53 and OIS, we finally asked whether the COX-2-p53 interaction is involved in the COX-2-mediated neoplastic transformation. The data showed that both COX-2-FL/H-RAS^V12^- and COX-2-NT/H-RAS^V12^-transfected MEFs formed foci easily on the soft agar, whereas MEFs transfected with COX-2-CT/H-RAS^V12^ rarely formed foci on the soft agar (Fig. 7a). In addition, the COX-2-FL/H-RAS^V12^- and COX-2-NT/H-RAS^V12^-transfected MEFs formed tumors easily in all injected sites of nude mice (Fig. 7b–d). These results demonstrate the critical role of the p53-interacting region of COX-2 to induce transformation, suggesting that the COX-2-p53 interaction is involved in the COX-2-mediated neoplastic transformation.

On the other hand, both COX-2/H-RAS^V12^- and S516Q/H-RAS^V12^-transfected MEFs formed foci easily on the soft agar with equal efficiency in MEFs (Fig. 7e). In addition, both COX-2/H-RAS^V12^- and S516Q/H-RAS^V12^-transfected MEFs showed equal efficiency to form tumors in nude mice (Fig. 7f–h). These results suggest that the COX-2 catalytic activity is not involved in the COX-2-mediated neoplastic transformation, supporting again our conclusion that the catalytic activity is not involved in the COX-2-mediated inhibition of pro-senescent function of p53 and OIS.

## Discussion

Accumulating in vivo evidence indicates that COX-2 is deeply involved in tumorigenesis, but the molecular basis is not fully understood. In this study, we found that COX-2 inhibits the pro-senescent function of p53 under oncogenic RAS activation, through which it prevents OIS and induces neoplastic transformation (Figs. 1–3). We also found that COX-2 inhibits the pro-senescent function of p53 by protein–protein interactions, through which it prevents OIS and induces transformation (Figs. 4–7). These findings strongly suggest that the oncogenic property of COX-2 is closely related to its ability to inactivate p53 under strong mitogenic signals, and that aberrant activation of the COX-2/a mitogenic oncogene combination can be a potent driving force for tumorigenesis. Actually, COX-2 is aberrantly overexpressed in most human cancers^19^, and mitogenic oncogenes are also activated in most human cancers^1,2^. In addition, OIS is mainly observed in cultured normal cells and premalignant lesions of animals^3,9–12,15^. Therefore, our findings also suggest that COX-2 can contribute to early stages of tumorigenesis including initiation and promotion by inhibiting OIS in normal and premalignant cells.

It has been reported that *COX-2*(+/+) and *COX-2*(−/−) MEFs show equal efficacy in the *EJ-ras/SV40*-induced transformation^39^. Based on our findings, this may be the result of SV40 inactivation of p53 regardless of the presence or absence of COX-2. In addition, COX-2 overexpression has been shown to fail to induce transformation in several normal and immortalized cells^35–38^. Based on our findings, concomitant expression of oncogenic RAS would have been able to induce transformation at least in rodent cells. Moreover, there are reports that *K-RAS*^*G12D*^*/COX-2* transgenic mice show increased pancreatic tumor incidence compared to *K-RAS*^*G12D*^ mice, which is attributable to COX-2-mediated inflammation and Notch signaling^46,47^. Based on our findings, however, this may also be the result of COX-2 inactivation of p53 by protein–protein interactions.

The role of COX-2 in cellular senescence has been in debate. COX-2 siRNAs slightly reduced H_2_O_2_-induced SA-β-gal activities in hTERT-immortalized HDFs, suggesting that COX-2 mediates senescence^48^. On the contrary, cisplatin-induced SA-β-gal activities decreased in COX-2-overexpressing CNE1 cancer cells and increased in *COX-2*(−/−) murine fibroblasts, suggesting that COX-2 inhibits senescence^49^. Here, our data clearly demonstrate that COX-2 acts as an anti-senescence factor. COX-2 prevents p53- and H-RAS^V12^-induced senescence in normal murine and human cells (Figs. 1 and 2). Since p53 plays a central role in senescence mediated by the DNA damage response (DDR) pathway^13^, COX-2 might prevent other DDR-mediated senescence as well as OIS.

Genetic models for malignant transformation provide a powerful tool to study the mechanisms of tumorigenesis. p53 inactivation is a critical step in many transformation models, which has usually been achieved by SV40 LT, HPV E7, Mdm-2, p53 mutants, or *p53* deletion^7,8,50^. We here propose COX-2 as a useful tool for p53 inactivation in the *p53*(+/+) models because COX-2 has several advantages. First, since *COX-2* is a known proto-oncogene, the use of COX-2 is clinically relevant. In the case of SV40, it is controversial whether it is involved in human tumorigenesis^51^. Second, COX-2 is overexpressed in most human cancers, making it a good choice for studying tumors of various organs. In the case of HPVs and Mdm-2, they are mainly involved in genital cancers and sarcomas, respectively^52,53^. Third, COX-2 has a strong capacity to inactivate p53 and induce transformation, as evidenced by the results that COX-2 inhibits the pro-senescent function of p53 and OIS completely (Figs. 1 and 2) and induces transformation as efficiently as SV40 LT (Fig. 3).

The pro-inflammatory catalytic activity of COX-2 is thought to be involved in tumorigenesis by regulating apoptosis, proliferation, angiogenesis, invasion, or metastasis^33,34^. Intriguingly, we here found that COX-2 inhibits the pro-senescent function of p53 through the p53-interacting region (amino acids 1–126) that lacks the catalytic domain, which is responsible for OIS prevention and transformation (Figs. 6 and 7). Compatible with this finding, we have reported that COX-2 inhibits the pro-apoptotic function of p53 through the p53-interacting region^44^. All these findings suggest that COX-2 can contribute to tumorigenesis by both catalytic activity-dependent and -independent mechanisms, expanding the scope of tumorigenic mechanisms of COX-2. Indeed, COX-2 has a binding ability to proteins or peptides^54–56^. Therefore, it may be possible that COX-2 contributes to malignant transformation through binding to not only p53 but also to other as-yet unidentified molecules.

We here also propose the COX-2-p53 interaction as a molecular target for the development of preventive or therapeutic drugs in COX-2-mediated tumors. Although specific inhibitors targeting the COX-2-p53 interaction have not been developed until now, it has been reported that SC-58125 and flurbiprofen induce conformational changes in the COX-2 molecule, especially in the membrane-binding domain (MBD, amino acids 59–111) (Fig. 6a), by binding to the active site of COX-2^57,58^. Therefore, it may be possible that known COX-2 inhibitors, whether selective or nonselective, modulate COX-2-mediated tumorigenesis by not only inhibiting the catalytic activity or regulating other molecules^59^, but also by affecting the COX-2-p53 interaction by inducing conformational changes in the p53-interacting region (amino acids 1–126).

It is still uncertain how the COX-2-p53 interaction inhibits the pro-senescent function of p53. According to our data, COX-2 overexpression did not reduce the p53 protein amount (Figs. 1c,f and 2c,f), suggesting that COX-2-p53 interactions do not inhibit the p53 function by p53 degradation. Instead, we found that COX-2 co-localizes with p53 in the nucleus under oncogenic RAS activation (Fig. 5c), suggesting that COX-2 inhibits p53 function by physical interactions with p53 in the nucleus. Further research is needed to elucidate the action mechanisms of COX-2 in the nucleus.

In our data, both H-RAS^V12^ transfection and COX-2/H-RAS^V12^ transfection induced nuclear translocation of COX-2 (Fig. 5c). Nevertheless, the H-RAS^V12^ transfection induced senescence, whereas the COX-2/H-RAS^V12^ transfection prevented senescence (Fig. 1). We think this phenomenon is because the amount of endogenous COX-2 in H-RAS^V12^-transfected MEFs was not sufficient to overcome the pro-senescent activity of p53, whereas the amount of COX-2 in COX-2/H-RAS^V12^-transfected MEFs was sufficient to overcome the p53 activity. Consistent with this idea, the amount of p53 and COX-2 precipitated by an anti-COX-2 and an anti-p53 antibody was very small in H-RAS^V12^-transfected MEFs, whereas it was increased abundantly in COX-2/H-RAS^V12^-transfected MEFs (Fig. 5a, b).

Although many clinical studies have reported that COX-2 overexpression is correlated with poor prognosis and low survival rates in a variety of human cancers^19^, the mechanisms by which COX-2 is overexpressed in cancer tissues are poorly understood. Promoter demethylation^60^, small mutations, or gene amplification may be candidates. As a preliminary study, therefore, we analyzed COX-2 gene amplification in cancer tissues by using public database (https://www.cbioportal.org)^61,62^. The data showed that the COX-2 gene was amplified in a variety of human cancers, particularly in breast cancer (15.3%) (Supplementary Fig. S3a). In addition, survival rates tended to decrease in breast cancer patients with COX-2 gene amplification compared to patients without gene amplification (Supplementary Fig. S3b). These results suggest that gene amplification may be a mechanism of COX-2 overexpression, at least in breast cancer. Further research is needed to verify this possibility.

**Figure 8:**
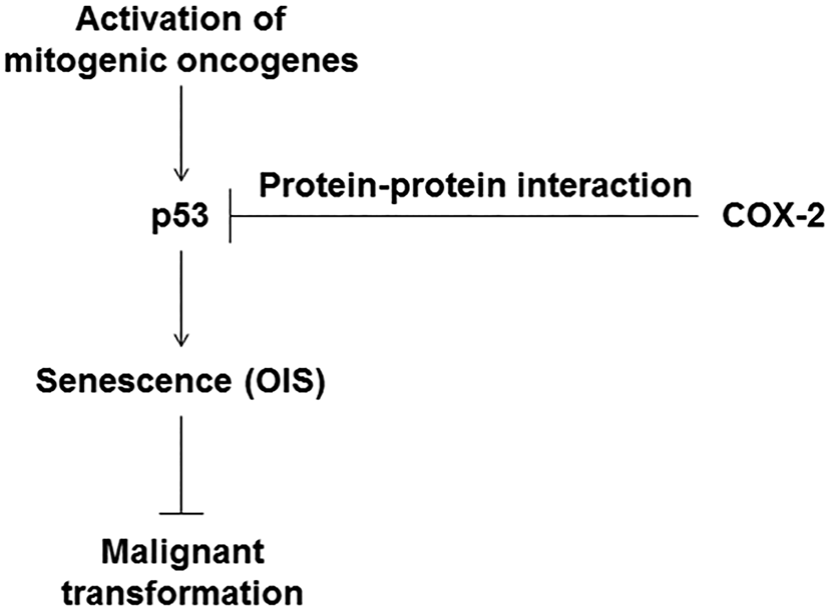
A proposed mechanism for COX-2-mediated malignant transformation.

Collectively, we here found that COX-2 inhibits the pro-senescent function of p53 by protein–protein interactions, by which it prevents OIS and induces transformation. These findings indicate that functional inactivation of p53 under strong mitogenic signals may be one of the mechanisms by which COX-2 contributes to tumorigenesis (Fig. 8). This study might contribute to our understanding of the molecular basis for the tumorigenic activity of COX-2.

## Supplementary Information

Supplementary Information
