# Supplementary material for: Cyclooxygenase-2 induces neoplastic transformation by inhibiting p53-dependent oncogene-induced senescence
Source: Sci Rep. 2021 May 10;11:9853. doi: 10.1038/s41598-021-89220-5 (PMC8110573; doi:10.1038/s41598-021-89220-5)

Cyclooxygenase-2 induces neoplastic transformation by inhibiting p53-dependent oncogene-induced senescence

Hyeon Ju Lee[1,†], So Ra Kim[1,†], Yu-Jin Jung[2], Jeong A. Han[1,*]

[1]Department of Biochemistry and Molecular Biology, Kangwon National University School of Medicine, Chuncheon, South Korea

[2]Department of Biological Sciences, Kangwon National University, Chuncheon, South Korea

[†]These two authors contributed equally.

*Corresponding author:

Jeong A. Han, MD, PhD

Department of Biochemistry and Molecular Biology, Kangwon National University School of Medicine, Chuncheon, 24341, South Korea

Phone: +82-33-250-8832; Fax: +82-33-250-8807

E-mail: gshja@kangwon.ac.kr

Supplementary Figure S1

a

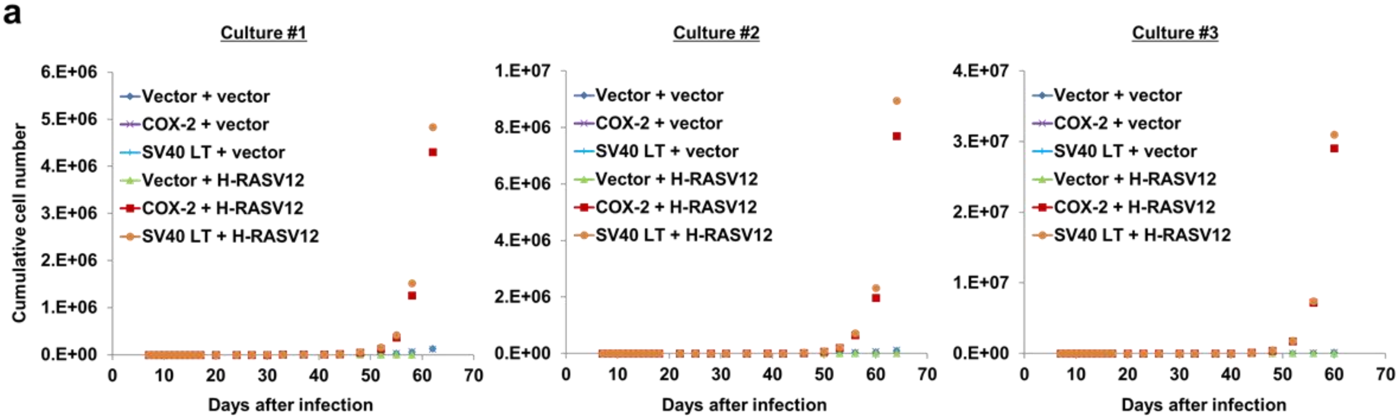

b

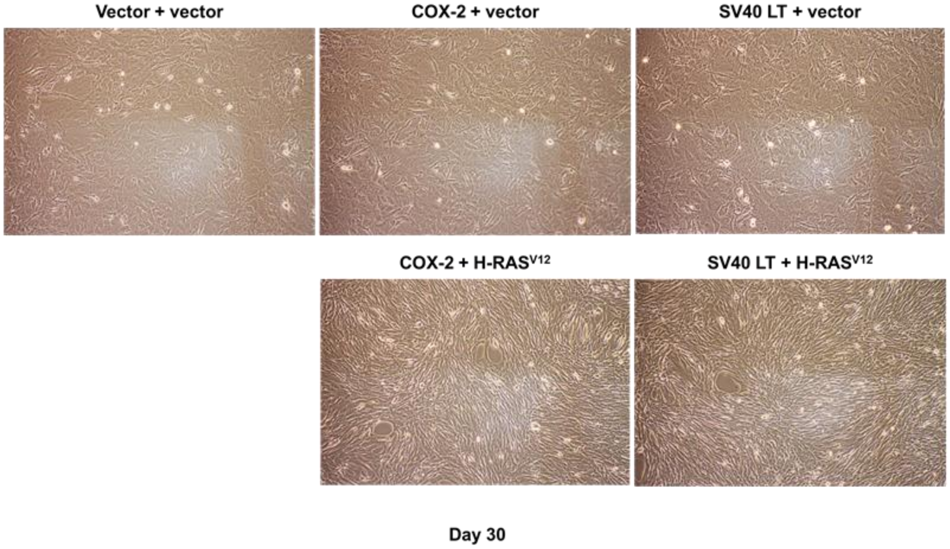

Supplementary Figure S1. Faster proliferation rates and altered morphology in COX-2/H-RAS^{V12}-transfected MEFs compared to Vector-transfected MEFs. a MEFs were infected with retroviruses as indicated. Then, 1 x 10^4 cells were seeded and the number of cells was counted for about 2 months. Representative culture data were shown. **b** On the 30th day after the infection, cell images were taken under a light microscope.

Supplementary Figure S2

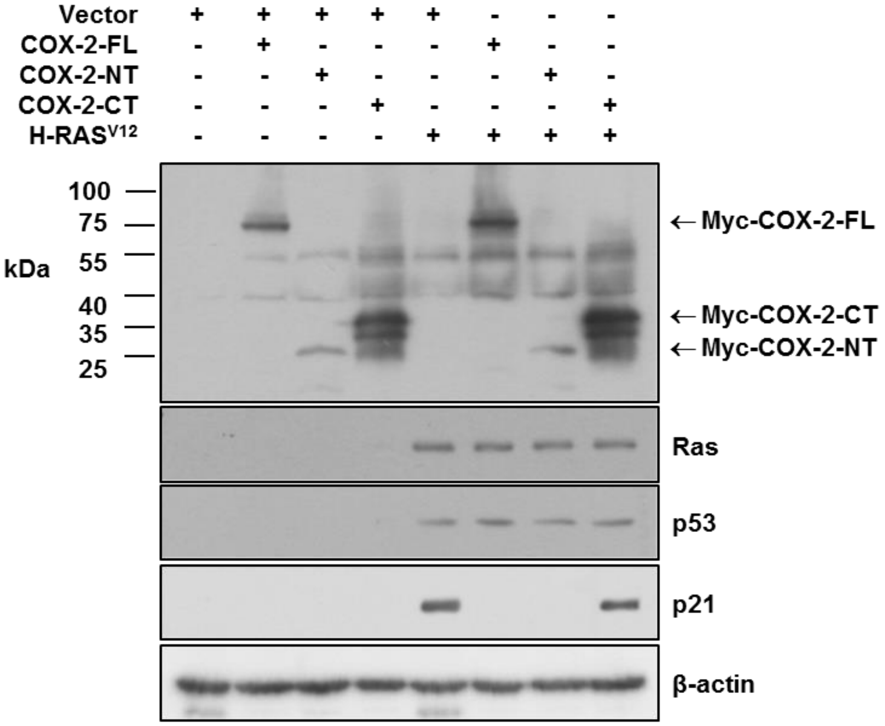

Supplementary Figure S2. MEFs were infected with retroviruses as indicated. Western blot analysis was performed using cell lysates. β-actin was used as a loading control.

Supplementary Figure S3

a

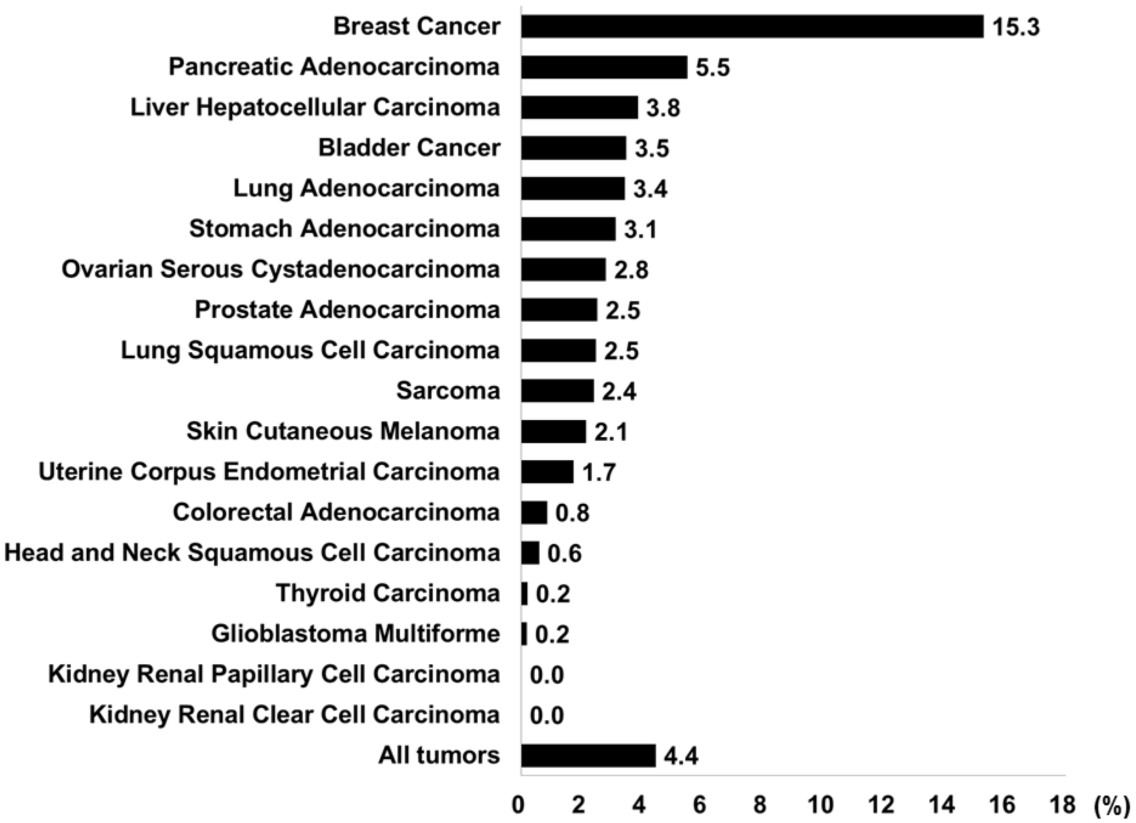

Supplementary Figure S3a. Amplification rates of the COX-2 gene in human cancers. The percentage of cancer samples with COX-2 gene amplification among cancers with more than 200 samples. Data downloaded from the c-Biopotal website (https://www.cbioportal.org).

Supplementary Figure S3

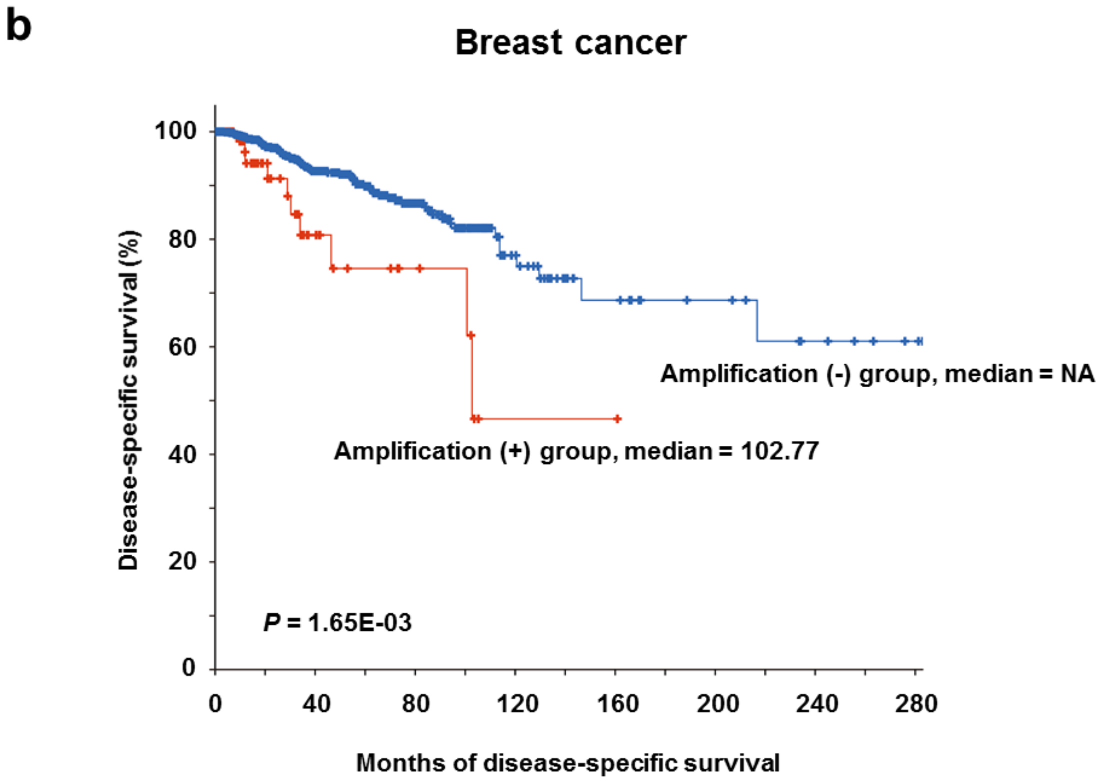

Survival Type	# Patients with data	p-Value
Disease-specific	1050	1.65E-03
Disease Free	929	6.56E-03
Progression Free	1068	0.0194
Relapse Free	2078	0.79
Overall	3049	0.931

Supplementary Figure S3b. Survival rates in breast cancer. Comparison of survival rates between the (+) and (-) groups of the COX-2 gene amplification in breast cancer patients. Logrank test. Downloaded from the c-Biopotal website (https://www.cbioportal.org).

Original Western Data Scan

Fig. 1c COX-2

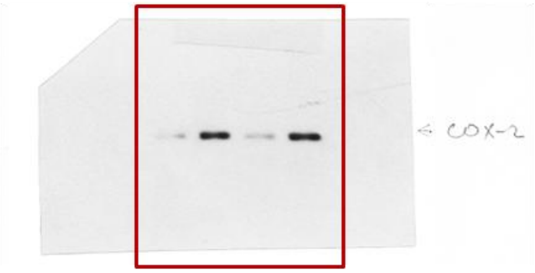

Fig. 1c Ras

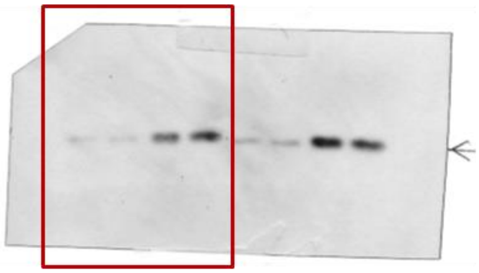

Fig. 1c p16

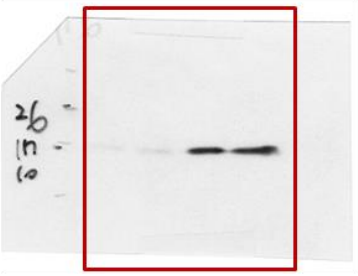

Fig. 1c p53

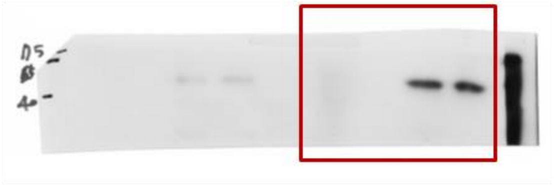

Fig. 1c beta-actin

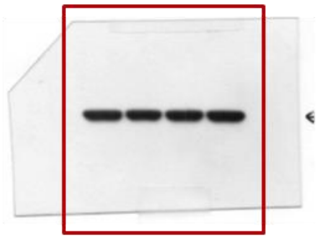

Fig. 1c p21

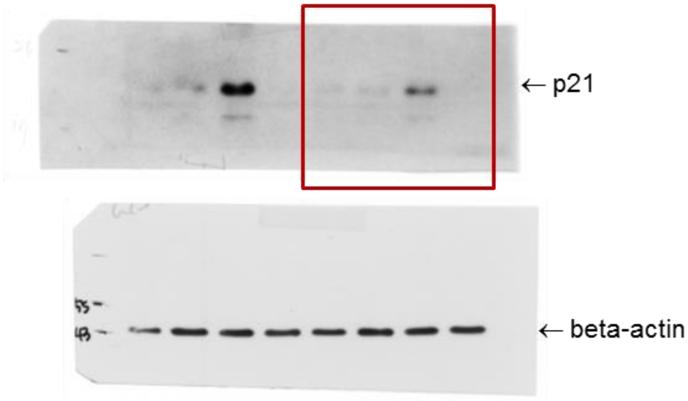

Blots were cut prior to hybridization with the 1st antibodies and therefore the whole length blots were not provided.

Original Western Data Scan

Fig. 1f COX-2

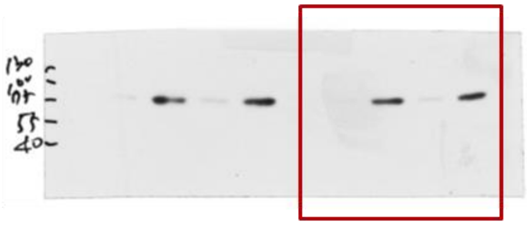

Fig. 1f Ras

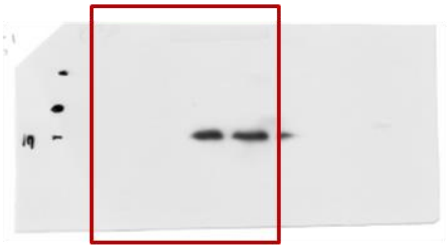

Fig. 1f p53

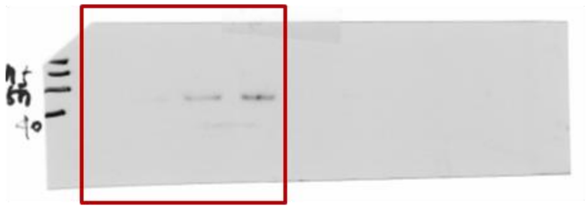

Fig. 1f p21

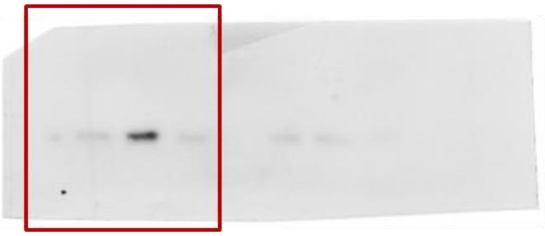

Fig. 1f p16

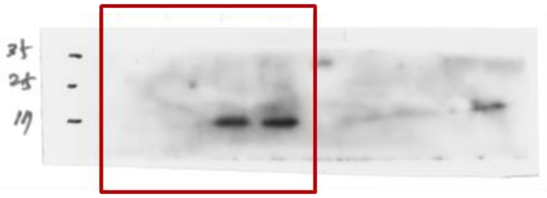

Fig. 1f beta-actin

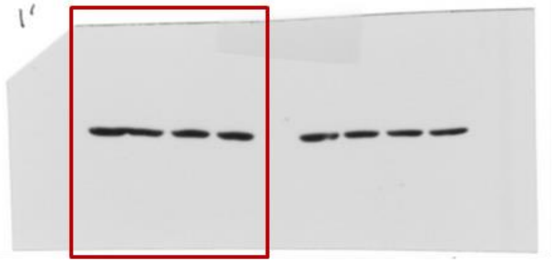

Blots were cut prior to hybridization with the 1st antibodies and therefore the whole length blots were not provided.

Original Western Data Scan

Fig. 2c COX-2

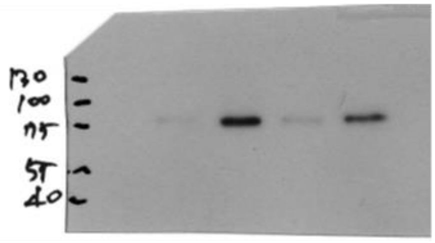

Fig. 2c p53

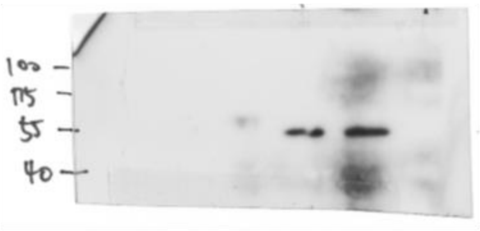

Fig. 2c p21

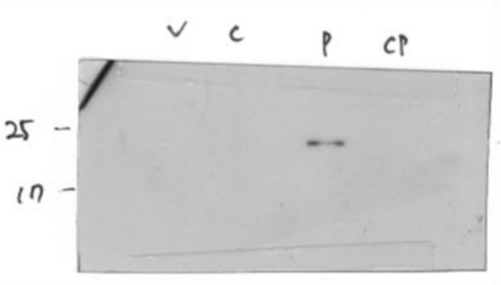

Fig. 2c beta-actin

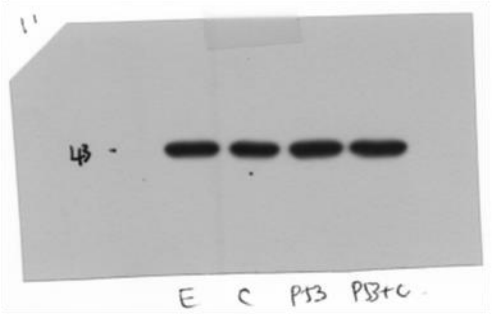

Blots were cut prior to hybridization with the 1st antibodies and therefore the whole length blots were not provided.

Original Western Data Scan

Fig. 2f COX-2

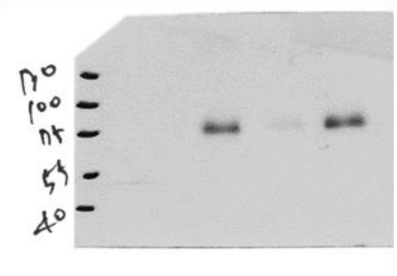

Fig. 2f p53

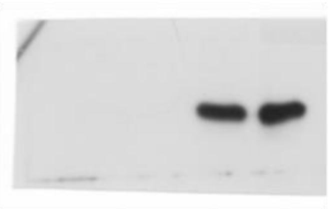

Fig. 2f p21

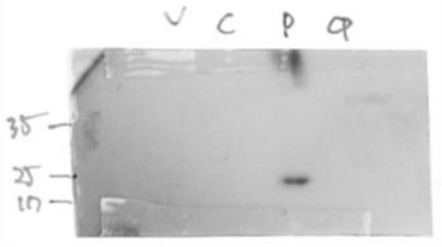

Fig. 2f beta-actin

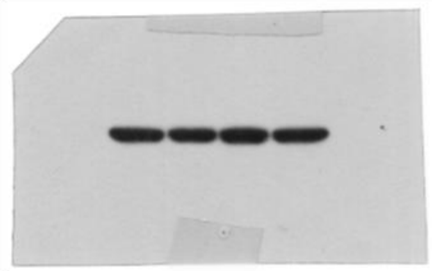

Blots were cut prior to hybridization with the 1st antibodies and therefore the whole length blots were not provided.

Original Western Data Scan

Fig. 5a p53

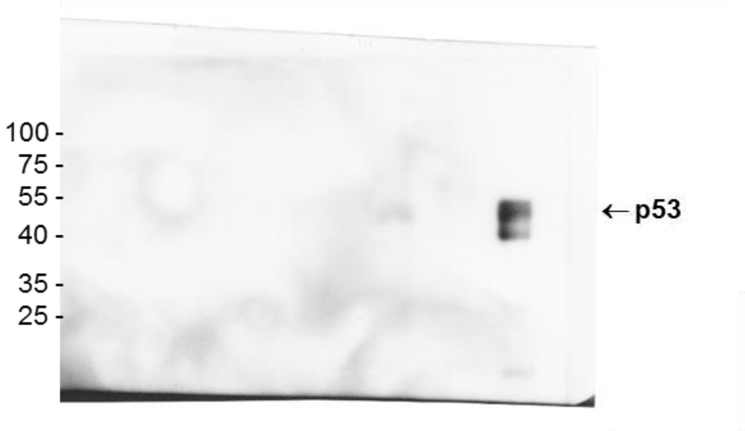

Fig. 5a COX-2

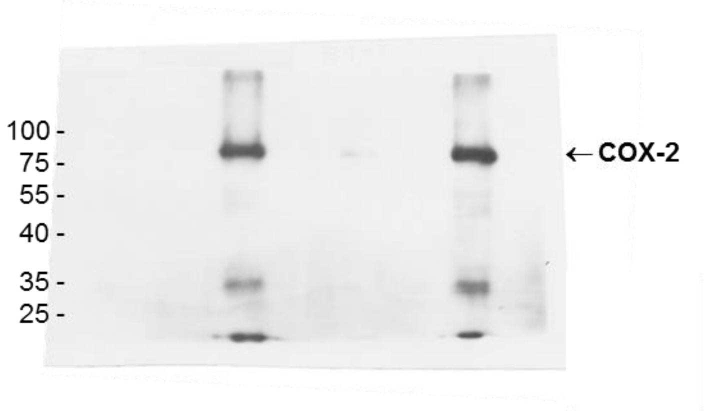

Original Western Data Scan

Fig. 5b COX-2

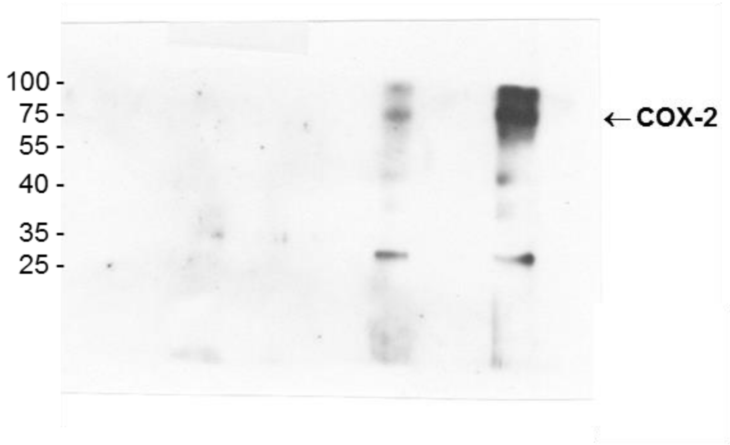

Fig. 5b p53

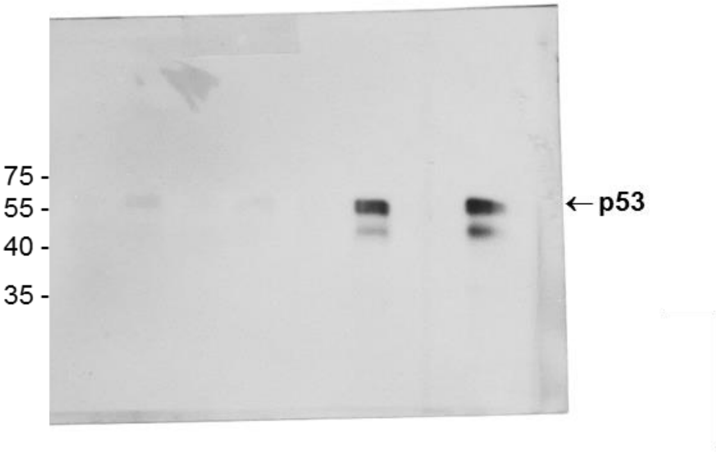

Original Western Data Scan

Supplementary Fig. S2. Myc

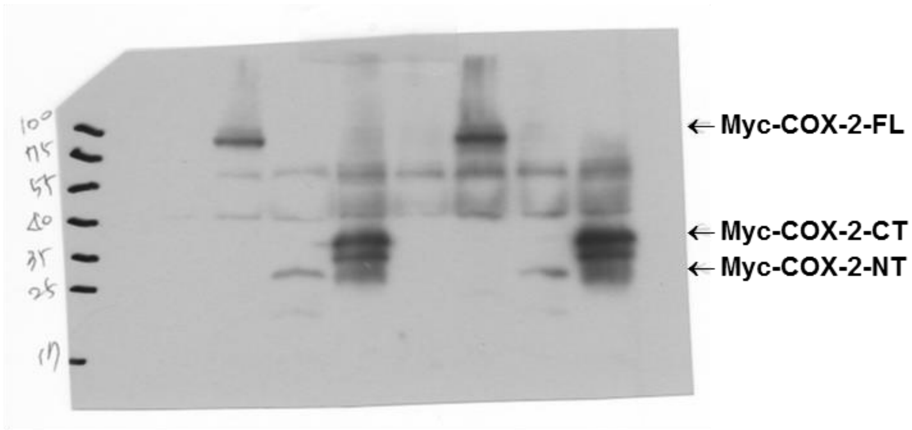

Supplementary Fig. S2. Ras

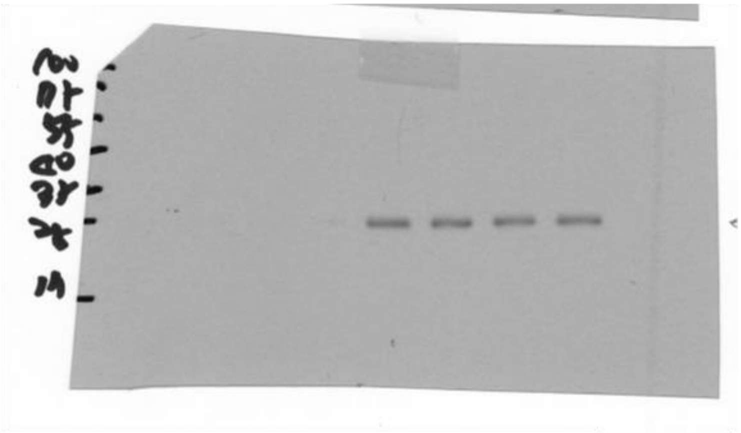

Original Western Data Scan

Supplementary Fig. S2. p53

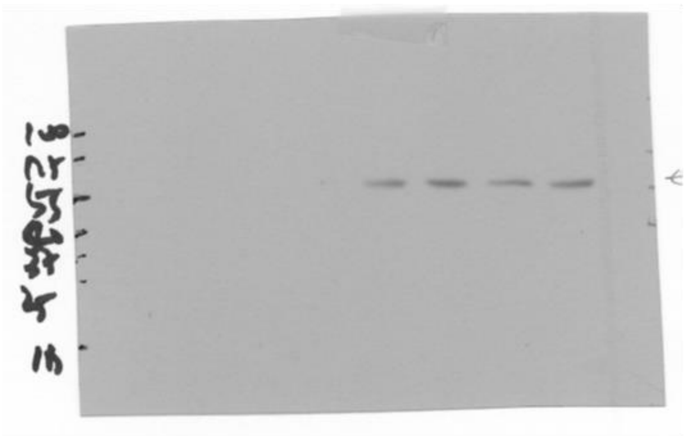

Supplementary Fig. S2. p21

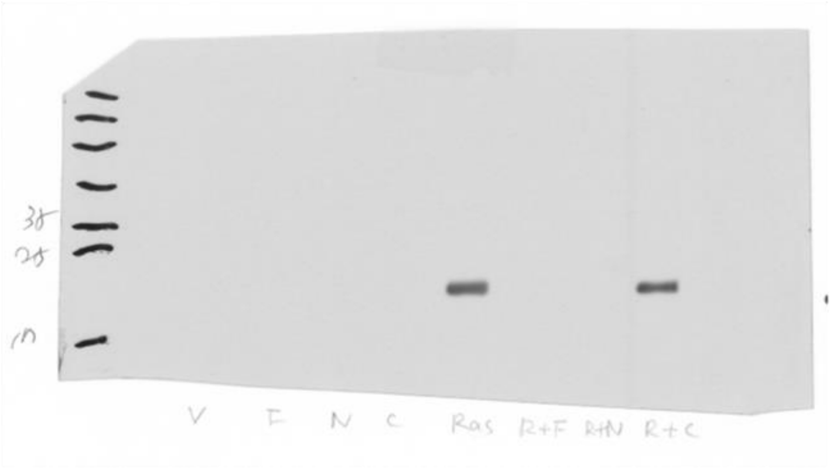

Original Western Data Scan

Supplementary Fig. S2. beta-actin

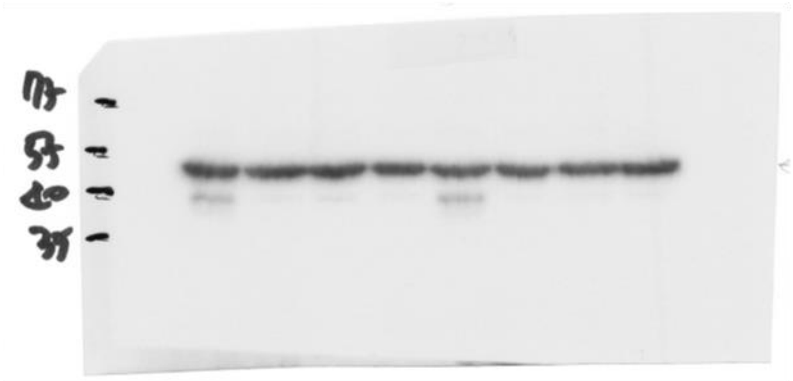

Supplement: Supplementary file 1 — Supplementary Information [file 41598_2021_89220_MOESM1_ESM.pdf]
